# Determination of odour concentration by TD-GC×GC–TOF-MS and field olfactometry techniques

**DOI:** 10.1007/s00706-017-2023-8

**Published:** 2017-07-13

**Authors:** Hubert Byliński, Paulina Kolasińska, Tomasz Dymerski, Jacek Gębicki, Jacek Namieśnik

**Affiliations:** 1 Department of Analytical Chemistry, Faculty of Chemistry, Gdansk University of Technology, Gdańsk, Poland; 2 Department of Chemical Engineering and Process, Faculty of Chemistry, Gdansk University of Technology, Gdańsk, Poland

**Keywords:** Gas chromatography, Odorous substances, Mass spectroscopy, Field olfactometry, Wastewater treatment plant, Oil refinery

## Abstract

**Abstract:**

Field olfactometry is one of the sensory techniques used to determine odour concentration, in atmospheric air, directly in emission sources. A two-dimensional gas chromatography with time of flight mass spectrometer (GC×GC–TOF-MS) allows performing the chemical characterization of various groups of chemical compounds, even in complex mixtures. Application of these techniques enabled determination of odour concentration level in atmospheric air in a vicinity of the oil refinery and the neighbouring wastewater treatment plant. The atmospheric air samples were analysed during a time period extending from February to June 2016. Based on the GC×GC–TOF-MS analysis and odour threshold values, the theoretical odour concentrations were calculated and compared with the odour concentrations determined by field olfactometry technique. The investigations revealed that higher values of odour concentration were obtained with the field olfactometry technique where odour analysis was based on holistic measurement. It was observed that the measurement site or meteorological conditions had significant influence on odour concentration level. The paper also discusses the fundamental analytical instruments utilized in the analysis of odorous compounds and their mixtures.

**Graphical abstract:**

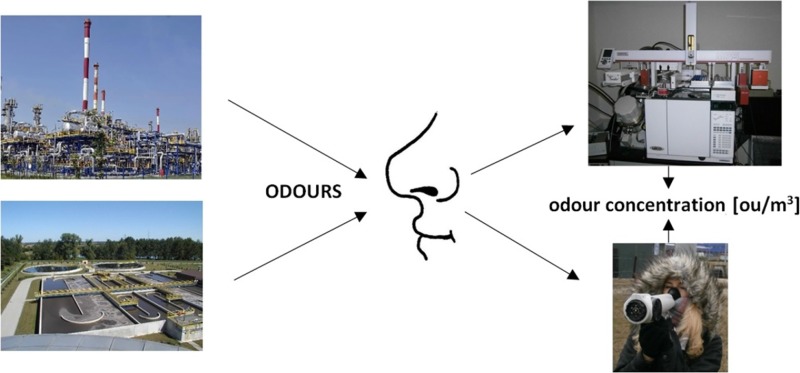

## Introduction

All forms of human activity, including urbanization of the areas located close to residential buildings, have significant impact on the air quality. Increasing amount of pollutants originate from municipal facilities such as landfills, recycling factories or wastewater treatment plants. Industrial plants including oil refineries, manufactories, breweries, distilleries and others also have significant impact on the introduction of chemical compounds from various groups to the atmosphere [1–4]. Air pollutants can have adverse effect on living organisms and abiotic part of the environment. Some of these can have carcinogenic properties, so they can be very dangerous to human life, especially at high concentrations. Among air pollutants, particular attention should be directed to all substances, which can be responsible for unpleasant aroma in air—odours [5–8]. Olfactory properties are exhibited by numerous chemical compounds, mainly volatile organic compounds, organic and inorganic nitrogen derivatives (ammonia or amines), inorganic sulfur compounds (hydrogen sulphide, methyl and dimethyl sulphide, dimethyl disulphide), aldehydes, ketones, esters, carboxylic acids, aliphatic and aromatic hydrocarbons, fatty acids, terpenes or chlorinated hydrocarbons [9–13]. Some of these compounds have very low odour threshold and, despite low concentration in atmospheric air, they can have significant impact on odour nuisance level in some areas.

To determine the chemical compounds present in various samples including atmospheric air, gas chromatography coupled with mass spectrometry detector (GC–MS) is often used [14–16]. This technique allows identification and quantification of the components in the odour mixtures. For complex samples, successful results can be obtained using two-dimensional gas chromatography (GC×GC). In this technique, two capillary columns with various stationary phases are connected. Usually, the first column is longer and less polar than the second one. GC×GC–TOF-MS technique has been successfully applied in various areas, including food analysis [17], environmental studies [18], petrochemicals analysis [19], and forensic analysis [20].

The olfactometry techniques are widely used to assess the sensory properties of many chemical compounds. During each analysis, the sense of smell is used as a measurement device. Among the sensory techniques used to determine the odour concentration, dynamic olfactometry enjoys an increasing popularity [21]. This method was described in the following standard: EN 13725 (2003) Air quality—determination of odour concentration by dynamic olfactometry, translated into Polish in 2007 [22]. To determine the odour concentration directly in emission sources, the field olfactometry technique is used [23]. Due to the possibility of determining small and quickly changing values of odour concentration, the field olfactometry (FO) technique is more and more frequently used to evaluate and monitor olfactory sensation, which can originate from various forms of human activities. The results from sensory evaluation allow identification of sources of odorants and estimation of the total odour emission in a particular measurement point at a particular period of time [24, 25]. One of the disadvantages of this technique is the necessity of an experienced panellists’ team, whose sensory sensitivity must be verified regularly. During long periods of time, the sensory sensitivity can deteriorate due to different factors, mainly olfactory fatigue. Field olfactometry technique finds increasing application in the evaluation of odour impact of different plants as the potential sources of odorous compound emission, for example, pig farms [26], mink farms [27], and sewage treatment plants [28–31].

Literature provides some papers concerning the comparison of different analytical techniques constituting the potential tools for odour quality evaluation. Capelli et al. [32] compared three instrumental techniques: GC–MS, dynamic olfactometry, and electronic nose technique as the potential tools allowing evaluation of the odour nuisance due to operation of a municipal landfill. Application of the GC–MS allowed determination and comparison of theoretical odour concentration, with the odour concentration determined by the dynamic olfactometry technique. As a result, there was a lack of correlation between the obtained results. Such situation was explained by the impossibility to measure all phenomena occurring in the odour mixture (odour synergism, odour attenuation) in case of determination of the theoretical odour concentration. The investigations carried out with the electronic nose instrument proved the ability of this technique to monitor changes of atmospheric air composition, for instance due to failure of industrial devices and installations. The authors emphasize that despite the lack of correlation between the results obtained with all three presented measurement techniques, each of them exhibits significant added value to the odour measurement problem as odour perception is a complex phenomenon. Another paper [8] presents the potentialities of several instrumental solutions aimed at characterization of the most important odorous substances present in atmospheric air, the emission of which is connected with the operation of a landfill. Among the presented techniques, there were gas chromatography–flame ionization detector and pulsed flame photometric detector (GC–FID/PFPD) as well as high-performance liquid chromatography (HPLC). The theoretical values of odour concentration were determined based on the concentration of particular chemical compounds and odour threshold values. In that paper, the authors proved the possibility of using this information to estimate the relative odour strength originating from the compounds present in atmospheric air.

Hansen et al. [33] utilized proton transfer reaction-mass spectrometry (PTR-MS) and dynamic olfactometry techniques to evaluate the effectiveness of a technology aimed at limitation of emission of odorous compounds generated by a pig farm. The investigations were carried out directly at the emission source (a mobile laboratory equipped with an olfactometer and spectrometer) as well as in the laboratory (air samples were collected into bags and olfactometric evaluation involved dynamic olfactometry technique). Comparison of the obtained results revealed substantial discrepancy between the odour concentrations obtained via dynamic olfactometry at the source as well as in the laboratory and the theoretical odour concentrations (named by the authors as “odour activity values”). A need for further investigation aimed at limitation of odour changes connected with air sampling into the bags was emphasized.

The purpose of this paper was to investigate the capability of two-dimensional gas chromatography coupled with time of flight mass spectrometer and field olfactometry techniques for characterization of the odour properties of atmospheric air in the part of Gdańsk City. In this area, a wastewater treatment plant and an oil refinery—one of the biggest industrial plants in the Pomeranian Voivodeship—are located. This research can show odour nuisance level over a period of 6 months and determine some factors, including atmospheric conditions, which can have significant impact on the odour nuisance. The paper contains a comparison of the theoretical odour concentrations obtained with the GC×GC technique with the odour concentrations acquired using field olfactometry. The reasons for the observed differences were discussed.

## Results and discussion

### Sensory analysis

Field olfactometry technique makes it possible to read out the values of “dilution to threshold ratio” (*D*/*T*). This parameter shows the ratio of the air stream that passed through the carbon filter (*V*_clean_) to the stream of odorous air (*V*_crude_). Based on the *D*/*T* values and Eq. (1), two values of dilution ratios were calculated: *Z*_YES_—dilution ratio corresponding to the first setting of *D*/*T*, when the odour became perceptible; *Z*_NO_—dilution ratio corresponding to the setting of *D*/*T* preceding the setting in *Z*_YES_.

1 $$Z = \frac{{V_{\text{clean}} + V_{\text{crude}} }}{{V_{\text{crude}} }} = \frac{{V_{\text{clean}} }}{{V_{\text{crude}} }} + 1 = \frac{D}{T} + 1.$$

To estimate the odour concentration, individual odour threshold estimate (*Z*_ITE_) was calculated as a geometric mean of the *Z*_YES_ and *Z*_NO_ values (Eq. (2)):

2 $$Z_{\text{ITE}} = \sqrt {Z_{\text{YES}} \times Z_{\text{NO}} } .$$

Odour concentrations were calculated as a geometric mean of *Z*_ITE_ from each measurement point.

In Table 1, the concentrations of odours at each measurement point (P1–P5) were presented. It can be observed that at two points—P2 and P3—the values of this parameter are significantly higher than in the remaining three measurement points. P2 and P3 points are located within the closest distance from two potential emitters of air pollution including odorous compounds—the wastewater treatment plant and the oil refinery. It could be one of the main reasons of higher values of odour concentration at these two points. Atmospheric conditions (air temperature, humidity, wind speed and direction) could also have a significant impact on the obtained results. Meteorological conditions during sampling are presented in Table 2. Based on the meteorological parameters shown in Table 2, it can be easily noticed that north and north-west winds were predominant during the time of olfactometric measurements. These winds could have moved air masses towards the points P2 and P3 located south with respect to the oil refinery, thus contributing to elevated odour concentrations at these measurement points.

**Table 1:** Average concentrations of odours at each measurement point during 5-month period of time/ou/m^3^

Measurement point	Month				
	II	III	IV	V	VI
P1	1.7	1.7	1.7	1.7	1.7
P2	3.1	3.5	4.5	5.1	6.2
P3	3.7	4.4	3.9	6.9	7.4
P4	1.7	1.7	1.7	1.7	1.7
P5	1.7	1.7	1.7	1.7	1.7

**Table 2:** Meteorological conditions during sampling in each month

Month	Air temperature/°C			Air humidity/%			Wind speed/m/s			Wind direction
	Min.	Max.	Abr.	Min.	Max.	Abr.	Min.	Max.	Abr.	
II	−3.3	9.1	2.9	64.6	96.4	80.5	2.3	14.5	8.4	NW
III	−1.0	15.8	7.4	57.0	96.5	76.8	3.5	17.3	10.4	NW/N
IV	0.8	20.7	10.8	46.7	96.4	71.6	4.9	17.4	1.,2	NW
V	6.1	28.3	17.2	38.2	97.0	67.6	3.7	12.2	8.0	N/SW
VI	7.7	32.3	20.0	27.7	97.4	62.6	3.1	12.6	7.9	N/NW

**Fig. 1:**
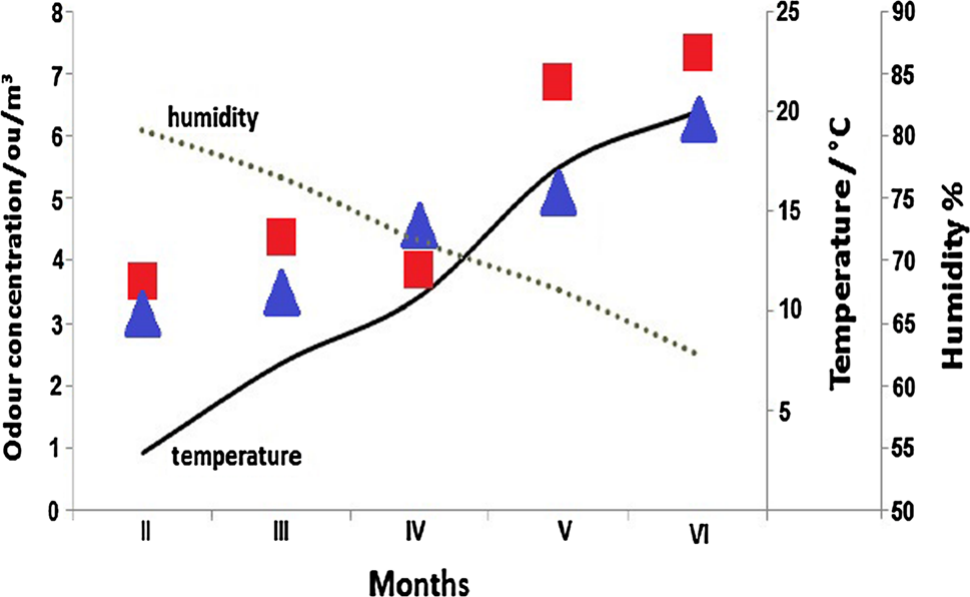
Comparison of odour concentration with respect to air temperature and humidity (*blue point* average concentration from P2, *red point* average concentration from P3)

The odour concentrations from P2 and P3 points were collected and compared in each month of sampling with the air temperature and humidity (Fig. 1). It can be observed that an increase in odour concentration occurs with an increase in air temperature and a decrease in air humidity. These parameters are very important from the viewpoint of determination of odour concentration in various times. In February–March–April, the minimum odour concentration was very similar, while in May and June these values were significantly different (Fig. 1). The maximum odour concentration was growing during the February–May period; in May and June these values were very similar.

### Chromatography analysis

Application of two-dimensional gas chromatography enabled identification of the main chemical compounds present in atmospheric air at the areas adjacent to the potential sources of these pollutants. Table 3 presents the main chemical compounds identified at one of the measurement points located in the vicinity of the oil refinery and the wastewater treatment plant. The identified chemical compounds were the most abundant ones on chromatograms. Moreover, the criteria of *S*/*N* > 10 was fulfilled in each case, which means that the substance appears above the limit of quantification (LOQ).

**Table 3:** Main chemical compounds identified at measurement point P3 in June

Name	First retention time/seconds	Second retention time/minutes	Similarity	*S*/*N*	Unit mass
Ethanol	320	2.37	952	18,043	27
2-Methylbutane	330	1.93	919	16,553	57
Pentane	340	1.94	891	8209.5	57
2,2-Dimethylbutane	355	1.94	921	16,415	57
Acetaldehyde	380	2.16	820	178.66	42
Hexane	410	1.98	947	17,862	86
2-Butenal	440	3.13	885	269.94	70
Benzene	465	2.17	964	60,419	78
Pentanal	500	2.27	939	7966.5	58
3-Ethyl-2,2-dimethylpentane	510	1.99	858	23,634	57
Toluene	635	2.46	936	1688.3	91
Ethyl butyrate	685	2.30	939	4338.0	71
Propyl propanoate	700	2.48	898	236.76	57
*m*-Xylene	830	2.53	713	31.124	91
Ethylbenzene	845	2.32	965	45,184	51
3-Ethylheptane	870	2.06	895	2850.5	57
*o*-Xylene	895	2.39	968	20,445	91
Propyl butyrate	895	2.30	851	144.69	88
Benzaldehyde	1015	3.52	971	13,159	106
Sabinene	1015	2.07	934	23,114	136
Pinene	1050	2.09	936	12,681	77
Hexadecane	1105	2.47	798	135.92	57
d-Limonene	1125	2.13	932	92,392	136
Terpinene	1155	2.18	884	11,991	93
Methyl heptanoate	1190	2.57	907	990.03	74
2-Ethyl-1-hexanol	1215	2.52	939	3844.1	112
5-Ethyl-2-methylheptane	1230	2.07	900	4667.6	71
Camphene	1235	2.15	954	13,609	121
Dodecane	1330	2.06	902	2112.8	71
Nonanal	1375	2.48	924	987.47	57
2-Ethylhexanoic acid	1430	4.40	886	1645.5	73
Tetradecane	1665	2.08	859	575.51	57
Hexadecanal	2020	2.42	890	107.21	57
Pentadecane	2135	2.06	874	304.14	57
1-Heptadecene	2595	2.10	829	23.197	31

**Fig. 2:**
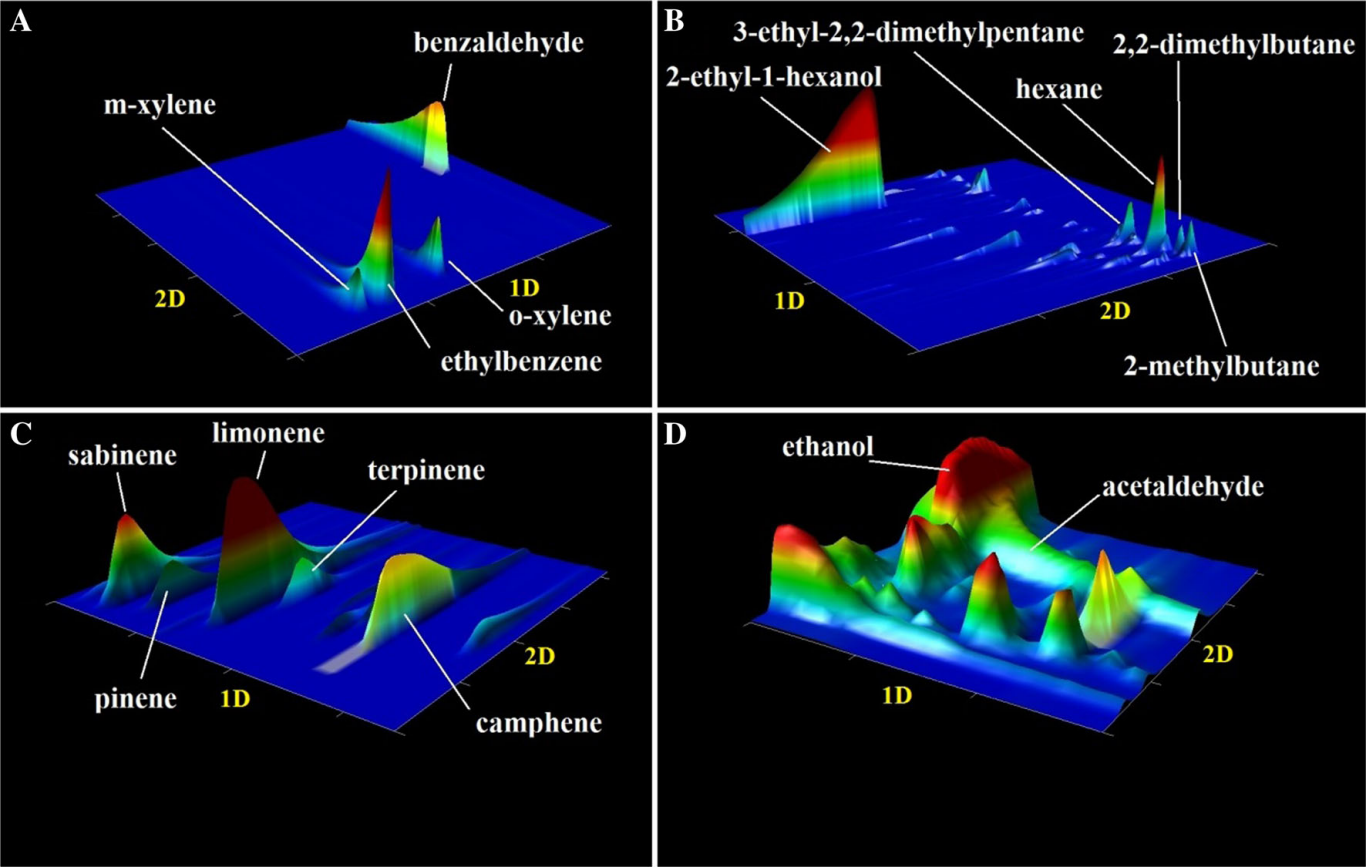
Chromatograms 2-D of the main compounds identified in the measuring point P3 in June

Figure 2 illustrates an exemplary 2-D chromatogram of the main compounds identified in point P3 in June. It can be observed that chemical substances belonging to different chemical classes have similar physicochemical properties, namely volatility, which can cause a co-elution in the first retention time. Therefore, application of the GC × GC system is important to separate chemical compounds with respect to two independent retention mechanisms based on volatility and polarity. In this case, it is possible to obtain a full separation. Some of these chemical compounds are responsible for malodour. Different odour properties such as odour intensity, hedonic quality, type of smell, concentration of each compound in the air and exposition time have significant impact on the level of odour nuisance in the area. Among the identified chemical compounds, many have characteristic odour and in many cases their smell is different despite being members of the same group of chemical compounds.

Dominant groups of chemical compounds identified in atmospheric air in the vicinity of the oil refinery and the neighbouring wastewater treatment plant are aliphatic hydrocarbons, aromatic hydrocarbons, aldehydes, ketones, terpenes and esters. Their presence is connected with the operation profile of the plants located in the neighbourhood of the investigated area and with the air masses transported from other parts of Gdańsk City.

### Determination of theoretical odour concentration and comparison with odour concentration

Theoretical odour concentration (*C*_od OT_) was calculated as a sum of chemical concentrations of all chemical compounds identified in one location (*C*_i_) and their odour threshold (*OT*_i_) ratio (Eq. (3)); N is the number of compounds.

3 $$C_{\text{od OT}} = \mathop \sum \limits_{i = 1}^{N} \frac{{c_{i} }}{{OT_{i} }}.$$

Table 4 shows the calculation of theoretical odour concentration at point P3 in June. A comparison of the theoretical odour concentration (*C*_od OT_) and the odour concentration determined by field olfactometry technique (*C*_od FO_) in each month is presented in Table 5.

**Table 4:** Calculation of theoretical odour concentration at point P3 in June

Chemical compound	Chemical concentration (*C*_i_) ± standard deviation (SD)/ppb	Odour threshold (*OT*_i_)/ppb [13, 34–36]	C_i_/OT_i_
Benzene	4.9 ± 0.5	2700	0.0016÷0.0020
Toluene	4.3 ± 0.4	330	0.0118÷0.0142
Ethylbenzene	5.7 ± 0.6	170	0.0300÷0.0370
*m*-Xylene	3.5 ± 0.4	41	0.0756÷0.0951
*o*-Xylene	6.7 ± 0.6	58	0.1051÷0.1258
*p*-Xylene	2.2 ± 0.2	380	0.0052÷0.0063
Limonene	6.6 ± 0.7	38	0.1552÷0.1921
α-Pinene	5.2 ± 0.6	18	0.2555÷0.3222
Pentane	4.3 ± 0.4	1400	0.0027÷0.0034
Hexane	8.2 ± 0.9	1500	0.0049÷0.0061
Heptane	3.6 ± 0.4	670	0.0048÷0.0060
Octane	4.9 ± 0.6	1700	0.0025÷0.0032
Nonane	6.1 ± 0.6	2200	0.0025÷0.0030
Decane	5.9 ± 0.7	620	0.0084÷0.106
Undecane	4.8 ± 0.5	870	0.0049÷0.0061
Dodecane	5.2 ± 0.5	110	0.0427÷0.0518
Acetaldehyde	4.4 ± 0.6	1.5	2.5333÷3.3333
Benzaldehyde	4.6 ± 0.6	42	0.0952÷0.1238
Sabinene	5.8 ± 0.7	75	0.0680÷0.0867
Phenol	5.1 ± 0.5	47	0.0978÷0.1191
Styrene	4.5 ± 0.5	47	0.0851÷0.1064
Biphenyl	6.1 ± 0.5	48	0.1167÷0.1375
1,3,4-Trimethylbenzene	4.4 ± 0.3	170	0.0241÷0.0276
1,3-Diethylbenzene	3.8 ± 0.5	70	0.0471÷0.614
1-Methylcyclohexane	4.8 ± 0.6	150	0.0280÷0.0360
2-Methylheptane	3.1 ± 0.5	110	0.0236÷0.0327
		Summary	3.8÷4.9

**Table 5:** Comparison of theoretical odour concentration (*C*_od OT_) and odour concentration (*C*_od FO_) for points P2 and P3

Location	II		III		IV		V		VI	
	*C*_od OT_	*C*_od FO_	*C*_od OT_	*C*_od FO_	*C*_od OT_	*C*_od FO_	*C*_od OT_	*C*_od FO_	*C*_od OT_	*C*_od FO_
P2	1.9	3.1	2.4	3.5	2.6	4.5	3.5	5.1	4.6	6.2
P3	2.1	3.7	1.7	4.4	1.9	3.9	4.3	6.9	4.4	7.4

A difference observed between the odour concentrations determined with GC×GC and field olfactometry can stem from several reasons. Chromatographic investigation provides separation of odorous mixture into particular components and thus yields qualitative and quantitative information about them. It is impossible to predict if these components exhibit odour-related mutual interaction, causing for example synergism (amplification of odour) or neutralization (attenuation of odour). In case of the olfactometric studies, where holistic measurement is performed, such effects can be observed and measured. The next factor, which can determine the obtained results, is the lack of unequivocal and universal odour threshold values. Literature provides several values of this parameter for particular compounds [13, 34–40], which result in underestimation or overestimation of the theoretical concentration. Another factor that determines the observed concentration difference is the limited number of chemical compounds identified and measured using GC×GC. Despite this fact, a relatively good correlation between the obtained results can be noticed that can be an evidence of a certain complementary character of the measurement techniques applied.

## Conclusion

Using two-dimensional gas chromatography technique allows quantitative and qualitative analysis of the main pollution present in atmospheric air at the areas adjacent to the potential odour emitters, such as the wastewater treatment plant and the oil refinery located in the south-east part of Gdańsk City. Based on this analysis, it is possible to calculate the theoretical odour concentration and to compare it with the odour concentration determined by field olfactometry technique. The determined theoretical odour concentrations varied from 1.7 to 4.6 ou/m^3^ depending on the measurement site (P2 and P3) and season of the year. In the case of theoretical determination of odour concentration based on the concentration of particular substances, it is not possible to take into account all phenomena connected with mutual odour amplification of particular components of an odour mixture. Both odour amplification—synergism—and odour attenuation can significantly change the strength of the odour sensed by humans. During the investigation described in the paper, it was impossible to identify all chemical compounds present in atmospheric air, which could have had an influence on the odour perception level. The aforementioned problems do not exist when the odour concentration is determined with field olfactometry technique. Olfactometric examination is a holistic approach; so it allows measurement of the entire odour mixture. The odour concentrations obtained with field olfactometry varied from 1.7 to 7.4 ou/m^3^ depending on the measurement site and season of the year. An increase in temperature is accompanied by intensification of decay processes occurring in wastewater treatment plants, which generate much higher amount of volatile compounds, including malodorous ones. In the case of oil refineries, the following phenomena take place: sweating of tanks at high temperature, leaking valves, leakages, and non-organized emission. These factors can contribute to enhanced emission of the odorous compounds.

The main chemical compounds identified during the investigations performed include aromatic and aliphatic hydrocarbons, aldehydes, ketones, terpenes, and esters. The compounds were present at the level of 1.4–8.7 ppb v/v depending on the site and the time of the measurement.

Weather conditions (wind speed, air temperature and humidity) were also monitored to show some correlation. Based on this research, an increase in odour concentration occurs with an increase in air temperature and a decrease in air humidity during the entire time period (5 months). High temperature increases the odour intensity and consequently the strength of the perceived odour. A higher content of water in the air can absorb some volatile compounds and the overall unpleasant odour is relatively smaller than in reality. Potentialities and limitations of particular techniques suggest that the most convenient solution is simultaneous application of a few methods, supplementary in character. Such approach allows complete illustration of the odour nuisance phenomenon, beginning with odour origination in different industrial or municipal facilities, through emission and immission processes, finishing with evaluation of exposure hazard of individuals or communities to unpleasant odour over a particular area.

## Experimental

### Sampling

The atmospheric air samples were collected during the 5-month period of time between February and June 2016 in the south-east part of Gdansk City. Every month, three measurement series were performed. The operation of the manufacturing plant (one of the biggest industrial plants in northern Poland) and the wastewater treatment plant located in this area can be connected with large emission of pollutants into the air.

**Fig. 3:**
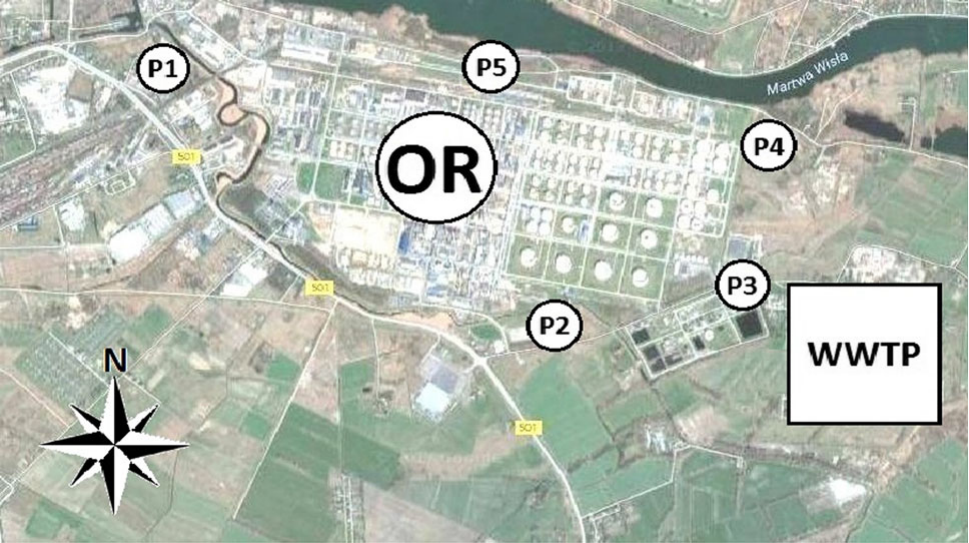
Location of measurement points; *OR* oil refinery, *WWTP* wastewater treatment plant [41]

The oil refinery located at this area has its own air monitoring stations in some of these points, where the concentration of the selected pollutants (methane, BTEX compounds, summary concentration of hydrocarbons and non-methane hydrocarbons) is measured. This area was selected for investigation due to close vicinity of two objects constituting a potential source of air pollution with odorous compounds—wastewater treatment plant and oil refinery. Localization of the measurement points was chosen in a way, which allowed revealing potential differences in ambient air quality around these objects. The measurement points P2–P4 were localized in the neighbourhood of the oil refinery and the wastewater treatment plant, whereas the measurement points P1 and P5 were in the vicinity of the oil refinery (Fig. 3).

A device called gas sampling system (GSS, Gerstel, Germany) was used for sample collection. This device allows collection of chemical compounds on the solid sorbent poly(oxy-2,6-diphenyl-1,4-phenyl) into the special tubes by pumping atmospheric air through these tubes. Before sampling, each tube was subjected to thermal desorption (temperature 280 °C, time of desorption 180 min) to remove potential pollution from previous studies. Between desorption and sampling, the sampling and analysis tubes were stored in dedicated containers at 15 °C. The volumetric flow rate of air through the tubes was 75 cm^3^/min and the sampling time equalled 30 min for each tube.

### Instrumentation

To determine the chemical compounds present in atmospheric air over the investigated area, the air samples were analysed using a two-dimensional gas chromatograph (Agilent Technologies, Palo Alto, CA, USA) equipped with a cryogenic modulator and coupled with a time-of-flight mass spectrometer (LECO Corp., St. Joseph, MI, USA). The column set consisted of a 30 m × 0.25 mm 0.25 μm primary column (1D) with Equity 1 stationary phase (Sigma Aldrich, USA) and a 2.0 m × 0.10 mm × 0.10 μm secondary column (2D) with Sol Gel Wax stationary phase (SGE Analytical Science, Australia). Separation of sample components was performed using the following optimized temperature programme for the primary GC oven:

- initial temperature of 40 °C,
- constant temperature for 1 min,
- temperature ramped at 10 °C/min to 90 °C,
- temperature ramped at 3 °C/min to 240 °C,
- constant temperature for 5 min,

and for the secondary GC oven:

- initial temperature of 45 °C,
- constant temperature for 1 min,
- temperature ramped at 10 °C/min to 95 °C,
- temperature ramped at 3 °C/min to 245 °C,
- constant temperature for 5 min.

The total analysis time was 65 min. Helium was used as a carrier gas (at a constant flow rate of 1.0 cm^3^/min). A modulation period of 5 s was employed with the cryogenic trap cooled to −196 °C using liquid nitrogen. The temperatures for the transfer line and the ion source were maintained at 250 °C. The detector voltage was set to 1600 V. Ions in the *m/z* = 40–500 range were analysed.

### Sensory analysis

Sensory analysis was performed using a Nasal Ranger field olfactometer (St. Croix Sensory, USA). The operation principle of this device is based on mixing odorous air and the air passed through a dedicated carbon filter in various proportions and evaluation of sensory properties of these mixtures by panellists. Before measurement series, the panellists (four people) were trained with regard to sensory evaluation, and the sensitivity of their sense of smell was checked according to a standard procedure employed to determine an individual sensitivity level of the sense of smell, developed by St. Croix Sensory, Inc. (St. Croix Sensory 2006, Minnesota). During each measurement series (3 days in each month), they were asked to indicate at which step of air dilution by odourless air the odour became perceptible. In each measurement point, each panellist conducted three evaluations at 10-min intervals. The measurements in each location were taken by all four panellists at the same time. Olfactometric evaluation was carried out simultaneously with a collection of the air samples intended for the analysis using two-dimensional gas chromatography.

### Data analysis

Data analysis was performed using the algorithm for peak deconvolution included in the Chroma TOF software (LECO Corp., USA, version 4.44). Tentative identification was accomplished through MS library search using the NIST 2011 and Willey 11 mass spectral library.
